# Age-related decline in spermatogenic activity accompanied with endothelial cell senescence in male mice

**DOI:** 10.1016/j.isci.2023.108456

**Published:** 2023-11-13

**Authors:** Manabu Ozawa, Hideto Mori, Tsutomu Endo, Yu Ishikawa-Yamauchi, Daisuke Motooka, Chihiro Emori, Masahiro Ikawa

**Affiliations:** 1 Laboratory of Reproductive Systems Biology, Center for Experimental Medicine and Systems Biology, The Institute of Medical Science, The University of Tokyo, Tokyo 108-8639, Japan; 2 Research Institute for Microbial Diseases, Osaka University, Osaka 565-0871, Japan

**Keywords:** biological sciences, Physiology, cell biology, Transcriptomics

## Abstract

Male fertility decreases with aging, with spermatogenic decline being one of its causes. Altered testis environment is suggested as a cause of the phenotype; however, the associated mechanisms remain unclear. Herein, we investigated the age-related changes in testicular somatic cells on spermatogenic activity. The number and proliferation of spermatogonia significantly reduced with aging in mice. Interestingly, senescence-associated β-galactosidase-positive cells appeared in testicular endothelial cell (EC) populations, but not in germ cell populations, with aging. Transcriptome analysis of ECs indicated that senescence occurred in the ECs of aged mice. Furthermore, the support capacity of ECs for spermatogonial proliferation significantly decreased with aging; however, the senolytic-induced removal of senescent cells from aged ECs restored their supporting capacity to a comparable level as that of young ECs. Our results suggest that the accumulation of senescent ECs in the testis is a potential factor contributing to the age-related decline in spermatogenic activity.

## Highlights

• The number and proliferation of spermatogonia significantly reduced with aging in mice
• SA-β-gal-positive cells appeared in testicular endothelial cell populations with aging
• Support from endothelial cells for spermatogonial proliferation decreased with aging
• Senolytics treatment of aged endothelial cells restored their supporting capacity

## Graphical abstract

**Figure:**
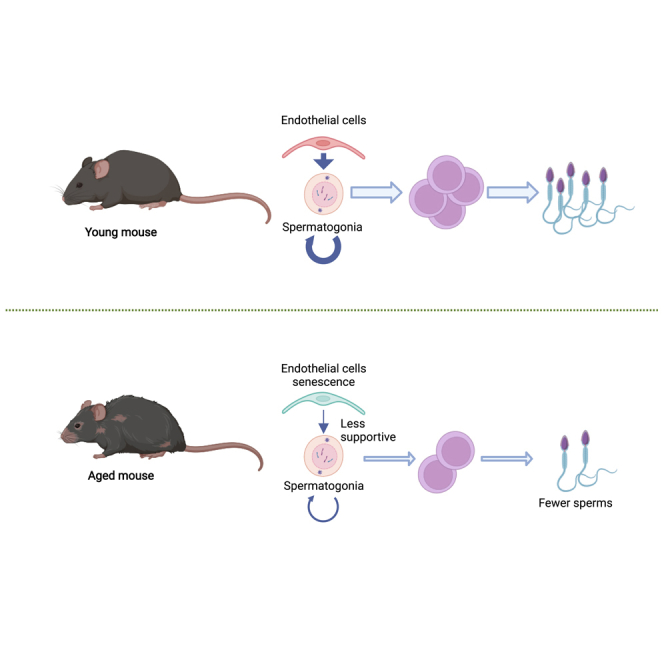

## Introduction

Age-related decline in fertility is widely observed in mammals, including humans. Currently, this adverse phenomenon has been demonstrated to be more pronounced in females. For example, mitochondrial dysfunction,^1^ abnormal epigenetics,^2^ and increased chromosomal missegregation^3^ frequently occur in oocytes with aging, thereby reducing the developmental competency of oocytes and ultimately lowering the pregnancy or delivery rates.^4^^,^^5^ In contrast, studies have shown that fertility potential declines with aging in males (e.g., a decrease in semen volume^6^ or sperm count^7^). The most commonly used criterion to define the adverse paternal age of fertility in human males is an age of >40 years at conception.^8^^,^^9^ In the mouse model, spermatogenesis or fertility has also been shown to decrease with aging; in a previous study, male fertility declined after the age of approximately 12 months, with an increase in the proportion of seminiferous tubules without germ cells.^10^ Furthermore, another study revealed that the number of spermatogonial stem cells (SSCs), which are considered the origin of all testicular germ cells, gradually decreases with aging.^11^

In the testis, spermatogenesis occurs inside the seminiferous tubules. Spermatogonia, including SSCs, are located in the outermost space of the seminiferous tubules and maintain their population through mitosis. Simultaneously, a group of spermatogonia is committed to differentiation by stimulating retinoic acid production by surrounding Sertoli cells.^12^^,^^13^ Germ cells that enter meiosis, also known as spermatocytes, undergo two rounds of meiotic divisions and differentiate into haploid spermatids. Then, spermatids undergo a period of maturation known as spermiogenesis and develop into flagellated, motile spermatozoa.

Although spermatogonia can proliferate *in vitro* (germline stem cells, GSC) for >5 years, which is longer than the average mouse life expectancy, a long-term culture of GSCs can lead to loss of their function as stem cells because long-term cultured GSCs cannot differentiate into spermatozoa after transplantation into the testis.^14^ However, when GSCs cultured *in vitro* for 2 years (the average lifespan of mice) are implanted in the testis of a young mouse, they could differentiate into spermatozoa and produce healthy offspring after fertilization.^15^^,^^16^ In addition, SSCs can proliferate for >3 years *in vivo* after being serially transplanted 9 times into the testes of young mice, where they can produce mature and competent sperm for producing healthy offspring.^10^ Thus, we hypothesized that age-related alteration in the testicular microenvironment is a cause of spermatogenic decline in aged males. However, the mechanisms underlying this phenomenon remain poorly understood.

In the present study, we showed that the proliferation of PZLF-positive spermatogonia declined in aged mice. Interestingly, our results clearly showed that senescent features were more pronounced in testicular endothelial cells (ECs), which are known to provide an environment essential for spermatogonial proliferation.^17^^,^^18^ Furthermore, we revealed that ECs from aged mice were less capable of supporting proliferation of GSCs, but the removal of senescent cells by senolytics restored their supporting ability. Our findings suggest a new insight that the age-related changes in ECs in the testis are a potential factor contributing to spermatogenic decline observed in older males.

## Results

### Aged male mice showed reduced fertility and lower sperm production

**Figure 1:**
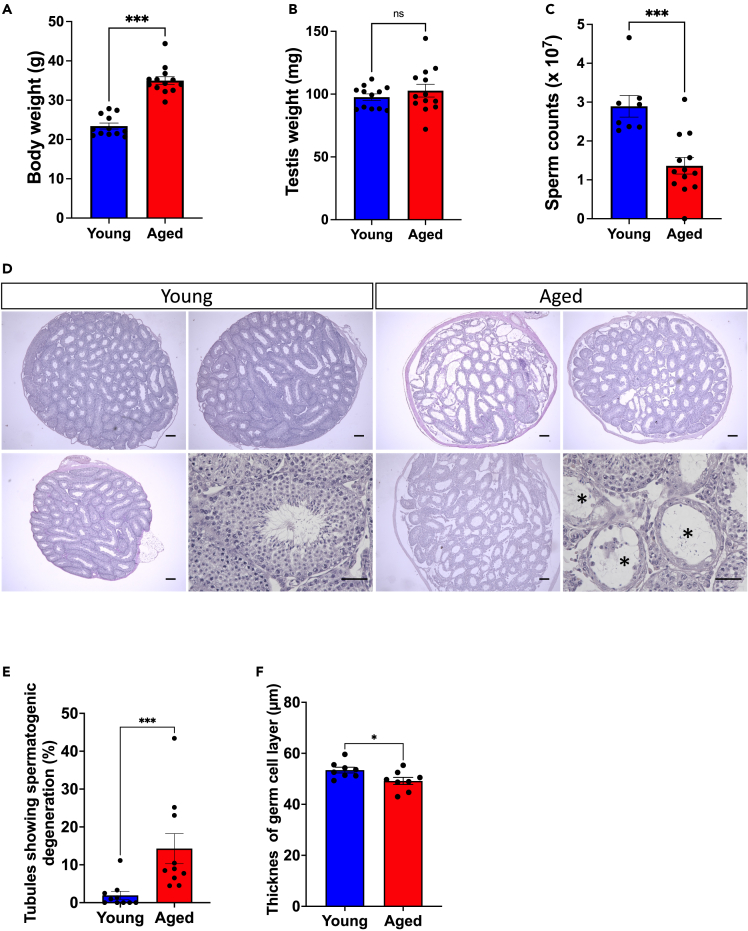
*Aged male mice show reduced fertility and lower sperm production* (A) Body weight of young and aged mice. The dots in the bar graph represent biological replicates of individual males (n = 12, young group; n = 13, aged group). ∗∗∗p < 0.001, indicating a significant difference. (B) Testis weight of young and aged mice. The dots in the bar graph represent biological replicates of individual males (n = 12, young group; n = 13, aged group). (C) Sperm counts in the cauda epididymis (×10^7^). The dots in the bar graph represent biological replicates of individual males (n = 8, young group; n = 13, aged group). ∗∗∗p < 0.001, indicating a significant difference. (D) Representative periodic acid-Schiff (PAS) and hematoxylin staining of the testis section from young or aged mice (three individuals of each age). Higher-magnification photos from an individual at each age are shown on the lower right panels. Seminiferous tubules with an asterisk (∗) represent spermatogenic degeneration. The scale bar denotes 100 μm. (E) Proportion of seminiferous tubules showing spermatogenic degeneration. The dots in the bar graph represent biological replicates of individual males (n = 10, young group; n = 10, aged group). ∗∗∗p < 0.001, indicating a significant difference. (F) Thickness of the germ cell layer in the seminiferous tubules indicates normal spermatogenesis. The dots in the bar graph represent biological replicates of individual males (n = 8, young group; n = 8, aged group). ∗p < 0.05, indicating a significant difference.

In this research, male mice aged 2 months or >2 years (designated as young or aged mice, respectively) were used. First, we conducted the mating test by housing male mice with young female mice to determine whether male reproductivity changes with aging. All four young male mice used for the mating test could consistently impregnate female mice. After 2 months of mating, the mean number of deliveries per head was 3.0 ± 0.7, and the mean number of pups per delivery was 7.7 ± 0.3. Conversely, only one of eight aged male mice could impregnate a female mouse, with the delivery occurring only once after 2 months of mating (Table 1). Next, the histological features of testes were compared between young and aged males. Although the body weight of aged males was significantly higher than that of young ones (Figure 1A, p < 0.001), the mean testis weight was comparable between the two groups (Figure 1B). Aged males had significantly lower sperm counts in the cauda epididymis (1.36 ± 0.21 × 10^7^ cells) than the young ones (2.89 ± 0.28 × 10^7^ cells) (Figure 1 C, p < 0.05). We also determined the correlation between body weight and sperm count and revealed no negative correlation (Figure S1). Regarding the histology of the testis cross section, some seminiferous tubules in aged males demonstrated a phenotype of spermatogenic decline, which is rarely observed in young males (Figure 1D, asterisk), and the proportion was significantly higher in aged males than in young males (Figure 1E, p < 0.001). Furthermore, germ cell layer thickness, which represents the activity level of spermatogenesis,^19^^,^^20^^,^^21^ in normal-appearing seminiferous tubules was lower in aged mice than in young ones (Figure 1F, p < 0.05). Taken together, fertility in aged male mice declined markedly, with lower spermatogenic activity with aging.

**Table 1:** Fertility of the young and aged male mice

Age	No. of males	No. of fertile males (ratio)	Total No. of delivery	No. of delivery per head	No. of pups per delivery
Young (2-month)	4	4 (100%)	12	3.0 ± 0.7	7.7 ± 0.3
Aged (>2-year)	8	1 (12.5%^a^)	1	0.13 ± 0.13∗	8 ± 0

a p < 0.05, indicating a significant difference.

### Aged testes exhibited spermatogenesis dysregulation and less active spermatogonial proliferation

**Figure 2:**
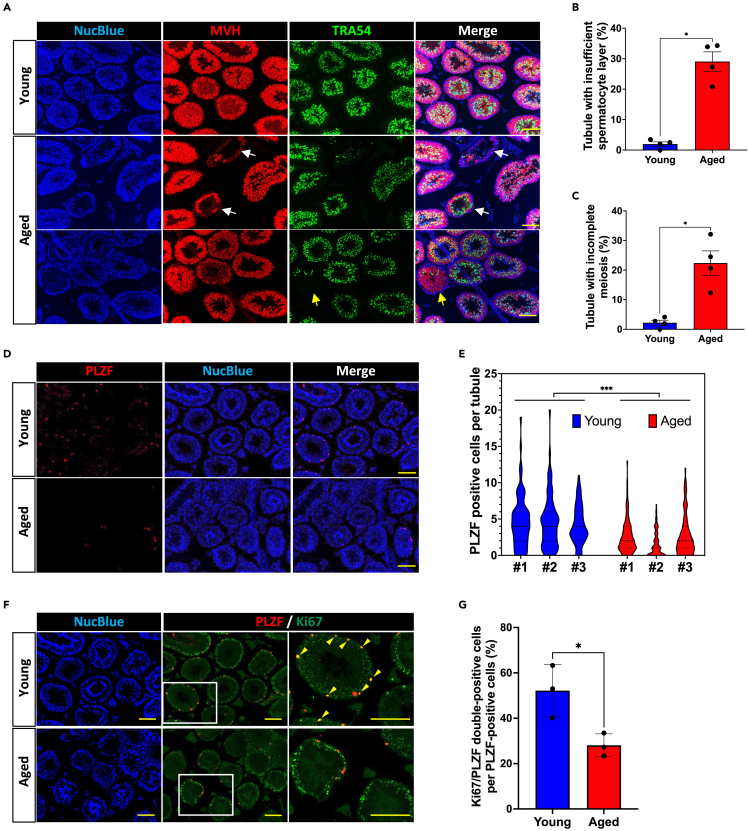
*Aged testes exhibit spermatogenesis dysregulation and less active spermatogonial proliferation* (A) Immunohistochemistry of testes from young and aged mice. Sections were stained with anti-MVH (a marker of germ cells) and anti-Haploid sperm cell-specific antigen (clone: TRA54) (a marker of haploid spermatid) antibodies. Nuclei were stained with NucBlue. White and yellow arrows depict the seminiferous tubules showing insufficient spermatocyte layers and incomplete meiosis, respectively. The scale bar denotes 100 μm. (B and C) Proportion of seminiferous tubules showing insufficient spermatocyte layers (B) or incomplete meiosis (C). Fourteen to twenty-five sections per individual male were used for counting, and the average was designated as one biological replicate. The dots in the bar graph represent biological replicates of individual males (n = 4 per group). ∗p < 0.05, indicating a significant difference. (D) Immunohistochemical analysis of PLZF in young and aged testes. Nuclei were stained with NucBlue. The scale bar denotes 100 μm. (E) Violin plots showing the number of PLZF-positive spermatogonia per seminiferous tubules in young or aged mice. Ninety-nine to a hundred and nine seminiferous tubules were observed for cell counting per individual male. Each violin plot shows data from individual mouse. Three males as biological replicates of each age were counted (n = 3, young group; n = 3, aged group). ∗∗∗p < 0.001, indicating a significant difference. (F) Immunohistochemical analysis of PLZF and Ki67 in young and aged testes. Nuclei were stained with NucBlue. Yellow arrowheads depict Ki67/PLZF double-positive proliferating spermatogonia. The scale bar denotes 100 μm. (G) The ratio of the number of Ki67/PLZF double-positive cells to the total number of PLZF-positive cells. The dots in the bar graph represent biological replicates of individual males (n = 3, young group; n = 3, aged group). ∗p < 0.05, indicating a significant difference.

Considering that spermatogenic defect was observed in aged testes, we assessed spermatogenic progression in further detail using immunohistochemistry. MVH (a pan-germ cell marker with a more robust expression in the cytoplasm of spermatocytes but relatively weaker expression in spermatogonia or spermatids) and TRA54 (a marker of haploid spermatids) were used for immunohistochemical staining. Multiple layers of MVH- or TRA54-positive cells were noted in almost all seminiferous tubules in young testes. Conversely, insufficient layers of MVH-high spermatocyte or TRA54-positive haploid spermatids (Figure 2A, white and yellow arrows, respectively) were observed in some tubules in aged testes. These proportions were significantly higher in aged testes than in young ones (Figures 2B and 2C; p < 0.05). Furthermore, aged mice had significantly fewer PLZF-positive spermatogonia per seminiferous tubule than young mice (Figures 2D and 2E; p < 0.001). Considering that spermatogonial cell numbers decrease with aging, we hypothesized that the mitotic activity of spermatogonia is lower in aged mice. We confirmed the proliferative potential of spermatogonia by assessing the expression of Ki67, a cell proliferation marker (Figure 2F). Approximately 50% (52.2% ± 6.7%) of PLZF-positive spermatogonia in young testes were positive for Ki67, whereas the proportion was reduced to 28.1% ± 2.9% in aged testes (Figure 2G, p < 0.05). Therefore, the testes of aged mice showed spermatogenesis dysregulation and less active spermatogonial proliferation.

### Testicular ECs in aged mice have a decreased capacity to support spermatogonial proliferation

**Figure 3:**
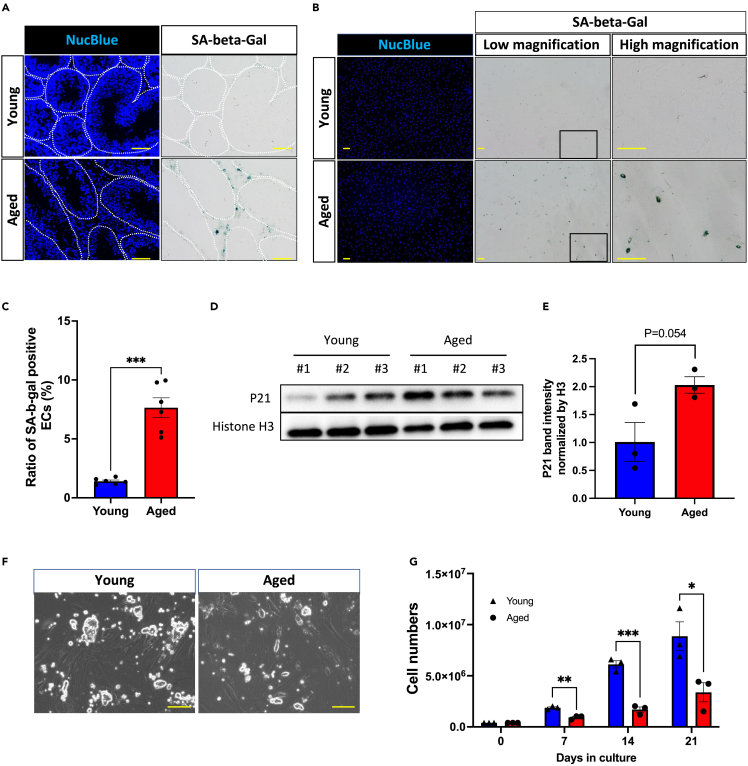
*Testicular endothelial cells (ECs) in aged mice have a decreased capacity to support spermatogonial proliferation* (A) Senescence-associated β-galactosidase (SA-β-gal) staining in young and aged mice. The white dotted line indicates the edge of the seminiferous tubules. The scale bar denotes 50 μm. (B) *In vitro* SA-β-gal staining of testicular ECs. The right panels indicate high-magnification images of the squares presented in the low-magnification images on the left panel. The scale bar denotes 200 μm. (C) Proportion of SA-β-gal-positive ECs. The dots in the bar graph represent biological replicates of individual males (n = 6, young group; n = 6, aged group). ∗p < 0.05 or ∗∗∗p < 0.001, indicating a significant difference. (D) Western blot showing p21 expression in ECs obtained from young or aged testes. Pan-Histone H3 was also analyzed as a loading control. Total protein was extracted from ECs for the analysis. Samples collected from three different individuals were added to each lane. (E) Quantifications of relative p21 protein expression using ECs from young and aged mice. The mean expression level of p21 in young ECs was designated as 1. The dots in the bar graph represent biological replicates of individual males (n = 3, young group; n = 3, aged group). The p value of the statistical difference between the age groups was 0.054. (F) Representative germline stem cell (GSC) colonies cocultured with ECs from young mice (left panel) or aged mice (right panel). The scale bar denotes 100 μm. (G) The mean proliferative rate of GSCs cocultured with ECs derived from young or aged mice. GSC numbers were counted every 7 days for 3 weeks. The dots in the bar graph represent biological replicates of individual males (n = 3, young group; n = 3, aged group). ∗p < 0.05, ∗∗p < 0.01, or ∗∗∗p < 0.001, indicating a significant difference.

Senescent cells accumulate in various tissues with aging and play a key role in driving age-related phenotypes and pathologies.^22^^,^^23^^,^^24^ Accordingly, the emergence and accumulation of senescent cells in the testes with aging were evaluated by performing senescence-associated β-galactosidase (SA-β-gal) staining in testis sections; SA-β-gal is a commonly accepted marker of cellular senescence both *in vitro* and *in vivo.*^25^^,^^26^^,^^27^ SA-β-gal-positive cells were rare in young testes but were common in aged testes. Interestingly, almost all SA-β-gal-positive cells were located adjacent to the outer side of seminiferous tubules, whereas nearly no positive cells were noted inside the seminiferous tubules with germ cells (Figure 3A). In the testes, ECs closely surround the outer region of the seminiferous tubules and play essential roles in controlling the proliferation or self-renewal of spermatogonia.^17^^,^^18^ Additionally, ECs in many tissues exhibit senescent phenotypes with aging.^28^^,^^29^^,^^30^ Thus, owing to aging, testicular ECs in mice might undergo senescence. Testicular ECs were isolated according to the previously reported method.^18^^,^^31^ We confirmed that almost all isolated cells were positive for CD34, which is a marker of testicular ECs (Figure S2A). First, SA-β-gal staining was performed to evaluate cellular senescence. Most ECs that were recovered from young testes were SA-β-gal negative, whereas a significantly higher number of ECs found in aged testes were SA-β-gal-positive (Figures 3B and 3C; p < 0.001). Next, the protein expression level of p21 (a CDK inhibitor protein and one of the common markers of cellular senescence) in ECs of each age was quantified and compared via western blot analysis. The mean protein expression of p21 tended to be higher in the ECs of aged testes than in those of young ones (Figures 3D and 3E; p = 0.054). Furthermore, the *in vitro* proliferation rate of ECs was significantly lower in aged mice than in young mice (Figures S3A and S3B; p < 0.05). Considering that testicular ECs are necessary for spermatogonial proliferation,^17^^,^^18^ we next determined age-related changes in the capacity of ECs to support spermatogonial proliferation. We evaluated the supportive capacity of ECs for spermatogonial proliferation through coculturing of GSCs and ECs, as reported previously.^17^^,^^18^ GSCs, which were developed from 7-day-old postpartum neonates in this study, proliferate to form typical grape-like colonies, and their morphology is distinct from that of other testicular somatic cells (Figure 3F). When cocultured with ECs that were recovered from young testes, GSCs exhibited sustained proliferation. However, when cocultured with ECs recovered from aged mice, the proliferation of GSCs was significantly inhibited (Figures 3F and 3G). Thus, the senescent phenotype was more pronounced in testicular ECs of aged mice than in those of young mice, and the ability of ECs to support spermatogonial proliferation diminished with aging.

### Transcriptome of aged ECs showed reduced proliferative features and increased expression of senescence-associated secretory phenotype (SASP)-related genes

**Figure 4:**
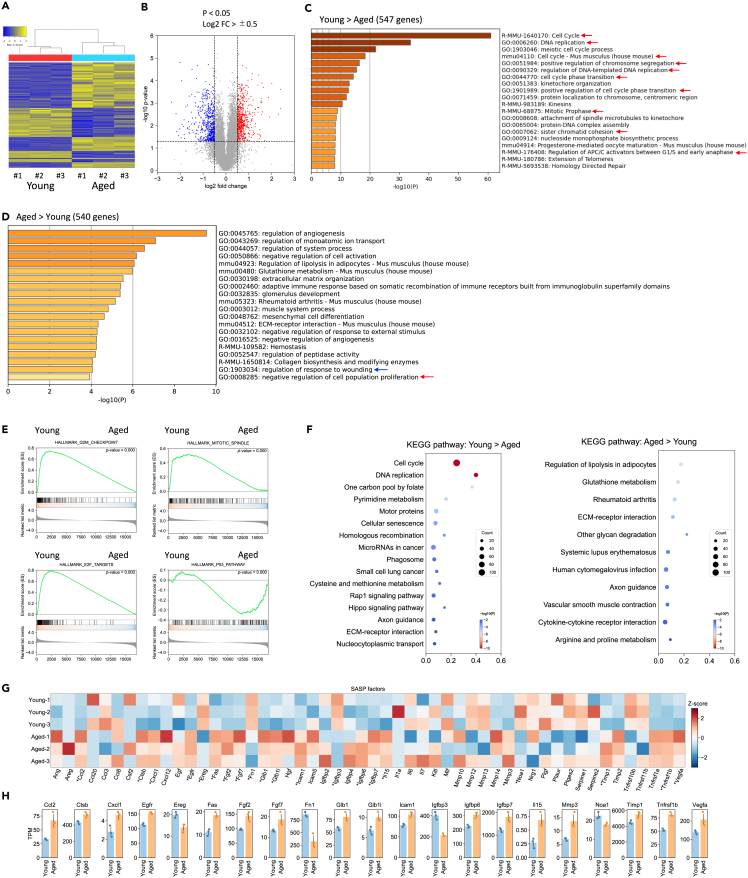
*Transcriptome of aged ECs shows lower proliferative features and increased expression of senescence-associated secretory phenotype (SASP)-related genes* (A) Heatmap showing differentially expressed genes in ECs from each age group. Clustering analysis revealed that gene expression patterns of mice of the same age were grouped into the same cluster (n = 3 in each age group, biological replicates). (B) Volcano plot showing the distribution of each gene expression level (x axis) and their p value (y axis). In ECs, 547 genes were downregulated (dots in blue), whereas 540 genes were upregulated (dots in red) with aging based on the cutoff criterion of log2 fold change of ≥0.5 or ≤ −0.5 and a p value of <0.05. (C and D) Gene Ontology (GO) analysis of the age-related downregulation (C) or upregulation (D) of genes in testicular ECs. Red arrows indicate GO terms related to cell cycle, cell division, or cell proliferation. (E) Gene set enrichment analysis (GSEA) of ECs derived from young and aged mouse testes. (F) KEGG enrichment analysis of upregulated and downregulated DEGs in ECs derived from aged and young testes. (G) Heatmap showing age-related alterations in the expression levels of senescence-associated secretion phenotype (SASP)-related genes.^33^ Gene expression patterns in mice of the same age were grouped into the same cluster (n = 3 in each age, biological replicates). ∗p < 0.05, indicating that the gene showed a significant difference. (H) The mean expression levels of SASP-related genes showing a significant difference in ECs between young and aged mice. Of the 19 genes with significant differences in expression, 15 were elevated in aged ECs.

Age-related transcriptomic alterations in testicular ECs were examined via next-generation sequencing. ECs cultured for 9 days post-harvest (dph) were used for the assays. Initially, RNA sequencing (RNA-seq) data were validated in a randomly selected set of 10 genes using quantitative real-time PCR (qPCR). Of these 10 genes, 8 showed statistically significant trends in both qPCR and RNA-seq analyses. Although the differences in the expression of the two genes, *Cdkn2a* and *Fst*, in ECs of each age were not statistically significant in qPCR analysis, the average expression of both genes was upregulated in aged ECs, consistent with the RNA-seq data. The corresponding p values for the two genes in qPCR analysis were 0.0732 and 0.0722 (Figure S4), suggesting that our RNA-seq results were reliable. Next, clustering analysis was conducted to confirm individual differences and reproducibility of transcriptome variations in young and aged mice (N = 3 in each age group). Gene expression patterns in ECs obtained from mice of the same age were grouped into the same clusters (Figure 4A). Next, differentially expressed genes (DEGs) with a log2 fold change of ≥0.5 or ≤ −0.5 and a p value of <0.05 were extracted (Figure 4B). Compared with young ECs, aged ECs had 540 upregulated and 547 downregulated genes. Interestingly, *GDNF* and *FGF2* (essential factors for the proliferation of SSCs) were not included in the DEG list, although the p value for FGF2 expression was <0.05 (Figure S5). Further, the DEG list was subjected to Gene Ontology (GO) analysis to determine the types of biological processes or signal transduction pathways that are altered in testicular ECs with aging. In the DEG set with age-related downregulation, GO terms related to cell proliferation or cell cycle progression, such as cell cycle, DNA replication, or cell cycle phase transition, were listed (Figure 4C, red arrows). In the age-related upregulation set, GO terms involved in the inhibition of cell proliferation, such as negative regulation of cell population proliferation (Figure 4D, red arrow) or wound healing as regulation of response to wounding (Figure 4D, blue arrow), in which senescent cells are actively involved,^32^ were included. In gene set enrichment analysis (GSEA), the number of several gene sets related to cell cycle and proliferation, such as G2M_checkpoint, Mitotic_spindle, and E2F_targets, decreased significantly in ECs obtained from aged mice compared with that in ECs obtained from young mice. Conversely, the P53_pathway gene set, which is related to mitotic arrest, was significantly accumulated in ECs obtained from aged mice, suggesting that the proliferative activity decreased in ECs obtained from aged mice (Figure 4E). Furthermore, to elucidate age-related changes in physiological and molecular pathways within ECs, we analyzed the DEG list using the Kyoto Encyclopedia of Genes and Genomes (KEGG). This analysis further confirmed the significant inhibition of pathways related to the cell cycle or DNA replication in aged ECs (Figure 4F). Next, we assessed the expression of SASP-related genes (one of the hallmarks of cellular senescence) based on the previously reported list.^33^ Of the 56 listed SASP-related genes, 19 showed a significant age-dependent difference in expression levels (Figure 4G, asterisk). Furthermore, compared with ECs obtained from young mice, 15 significant DEGs were upregulated in ECs obtained from aged mice (Figure 4H). Therefore, the ECs of mouse testes exhibited senescent characteristics with aging.

### Treatment of aged ECs with senolytics recovered their potential for supporting spermatogonial proliferation

**Figure 5:**
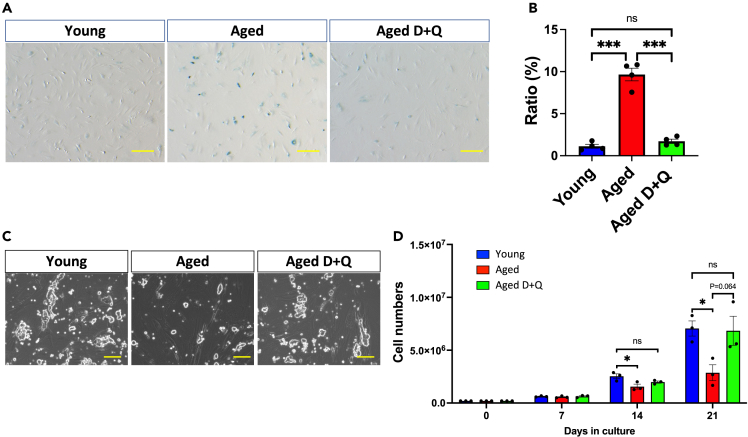
*Treatment of aged ECs with senolytics recovers their potential for supporting spermatogonial proliferation* (A) SA-β-gal staining of testicular ECs *in vitro* with or without dasatinib (50 nM) and quercetin (25 μM) (D + Q). The left and middle panels show ECs from young and aged mice, respectively, and the right panel shows D + Q-treated ECs from aged mice. The scale bar denotes 100 μm. (B) Proportion of SA-β-gal-positive ECs. The dots in the bar graph represent biological replicates of individual males (n = 4, young group; n = 4, aged group; n = 4, aged group treated with D + Q). ∗p < 0.001, indicating a significant difference. (C) Representative GSC colonies cocultured with ECs derived from young mice (left panel) or ECs derived from aged mice that were treated with (right panel) or without D + Q (middle panel). The scale bar denotes 100 μm. (D) The mean proliferative rate of GSCs cocultured with ECs derived from young or aged mice treated with or without D + Q. GSCs were counted every 7 days for 3 weeks. The dots in the bar graph represent biological replicates of individual males (n = 3, young group; n = 3, aged group; n = 3, aged group treated with D + Q). ∗p < 0.05, indicating a significant difference.

Removing senescent cells using senolytics reportedly mitigates age-related deleterious phenotypes such as diabetes,^34^ cardiac dysfunction,^35^^,^^36^ vascular hyporeactivity,^37^ and renal dysfunction.^38^ Thus, removing senescent cells from aged ECs might recover ECs’ capacity to support spermatogonial proliferation. Among the reported senolytics, the combination of the SRC/tyrosine kinase inhibitor dasatinib (D), which has been approved by the US Food and Drug Administration, and the natural flavonoid quercetin (Q) has been commonly applied to research as well as clinical trials (D + Q). Therefore, we determined whether D + Q treatment can remove senescent cells from ECs obtained from aged mouse testes. These ECs were first treated with D + Q for 6 days under *in vitro* culture; then, SA-β-gal staining was performed to compare the proportions of SA-β-gal-positive cells. We revealed that the ECs from aged testes that were treated with D + Q showed a significant decrease in the proportion of SA-β-gal-positive cells compared with untreated ECs (Figure 5A). Subsequently, the proportion of SA-β-gal-positive cells in ECs from aged testes was comparable to that in ECs from young testes (Figure 5B), suggesting that D + Q treatment successfully removed senescent ECs. Finally, we used the GSC and EC coculture system to determine whether ECs’ capacity to support spermatogonial proliferation recovered after removing senescent cells by D + Q treatment. When GSCs were cocultured with D + Q-treated ECs obtained from aged testes, the proliferative rate of GSCs was similar to that of ECs obtained from young testes, with no significant difference between young ECs and aged ECs treated with D + Q (Figures 5C and 5D). Therefore, the decreased spermatogenic potential observed in the testes of aged mice might partly be due to the impaired spermatogonial proliferation caused by the accumulation of senescent ECs.

## Discussion

In this study, we analyzed the effects of aging on male fertility. Results showed that the mitotic activity of spermatogonia was weakened in aged mice, and sperm counts were significantly decreased, resulting in a marked decline in fertility. In addition, the accumulation of senescent ECs in the testis was observed with aging. Interestingly, the *in vitro* culture model indicated that removing senescent ECs by senolytics treatment could restore their supporting capacity for spermatogonial proliferation. These results indicate a possibility that an age-related increase in the proportion of senescent testicular ECs could be a cause of the spermatogenic decline.

The sperm count has been reported to decrease in aging male mammals, such as rats^39^ and humans.^7^ A previous mouse study also showed that the number of spermatogonia decreases with aging.^11^ These results are consistent with our findings such as a significant decrease in the number of PLZF-positive spermatogonia in aged testes (Figures 2D and 2E) as well as a decrease in the proportion of Ki67-positive mitotic spermatogonia (Figures 2F and 2G). Although no stage-specific arrest of germ cell differentiation was observed, compared with young mice, the thickness of the germ cell layer reduced (Figure 1F), and the proportion of the seminiferous tubules with insufficient spermatocyte or spermatid layers increased in aged mice (Figures 2B and 2C). Thinning of the germ cell layer accompanied with reduced sperm counts resulting from decreased spermatogonial proliferation has been reported in various studies.^40^^,^^41^ Therefore, a reduction in the number of spermatogonia caused by their low proliferative activity could be a cause of the lower sperm counts observed in aged individuals.

Serial transplantation of SSCs into the testes of young mice allows the SSCs to continue spermatogenesis beyond the lifespan of mice.^10^ When SSCs are transplanted into aged mice, their ability to form spermatozoa is suppressed.^10^^,^^11^ In addition, single-cell RNA-seq results using human testis samples revealed that the changes with aging are more pronounced in somatic cells but less evident in germ cells.^42^ Thus, the properties of somatic cells in the testes tend to alter with aging, leading to alterations in the testicular environment. Likewise, our study showed that SA-β-gal-positive germ cells were rarely detected within the seminiferous tubules even in aged mice. However, the proportion of somatic cells located outside the seminiferous tubules markedly increased with aging (Figure 3A). Histologically, ECs are localized in this region.^17^^,^^18^^,^^31^ We observed that the proportion of SA-β-gal-positive ECs predominantly increased; additionally, the protein expression of p21, which inhibits cell proliferation and is highly expressed in senescent cells,^43^^,^^44^ increased in ECs obtained from aged mice. Furthermore, the expression of a set of genes related to proliferation or cell division decreased, whereas that of SASP-related genes, one of the characteristics of senescent cells,^33^ increased in ECs derived from aged mice. Therefore, testicular ECs undergo senescence with aging; however, future *in vivo* studies are warranted to determine the senescence of other testicular somatic cells, such as Leydig and myoid cells.

Testicular ECs, which are located outside the seminiferous tubules, regulate the proliferation and self-renewal of spermatogonia by secreting GDNF^17^ and FGF.^18^ ECs recovered from aged mice showed lower capacity to support GSC proliferation than those recovered from young mice. However, removal of senescent cells from ECs that were recovered from aged mice by D + Q treatment restored ECs’ ability for supporting GSC proliferation. Therefore, the accumulation of senescent cells in ECs suppresses spermatogonial proliferation. Moreover, this study showed no significant difference in GDNF mRNA or a slight (1.46-fold) increase in FGF2 mRNA expression in aged ECs compared with that in young ones (Figure S5). The expression of factors other than GDNF or FGF, which accelerate the proliferation of germ cells, may decrease with aging. In fact, the *in vitro* culture of GSCs requires MEFs as feeder cells in addition to GDNF and FGF. In B6 and 129 background mice, GSCs develop poorly even after supplementation with GDNF and FGF.^15^ Thus, other factors in ECs, in addition to GDNF and FGF, could play an important role in spermatogonial proliferation. Another possibility is that aged ECs produce factors that inhibit the proliferation of germ cells. ECs recovered from aged mice showed an elevated expression of proinflammatory cytokines related to SASP (Figures 4F and 4G). In mouse experimental autoimmune orchitis model, which exhibits a chronic physiological inflammation, the proliferation and differentiation of spermatogonia are reduced, resulting in spermatogenic defects.^45^ In addition, mitosis in GC-1 cells, a cell line originating from spermatogonia, can be arrested by adding the inflammatory cytokine TNF-α to the culture medium.^46^ Insights into the changes associated with the senescence of testicular ECs that affect the proliferative potential of spermatogonia should be analyzed in future research.

### Limitations of the study

We also note the certain limitations of this study. We determined the age-related alteration of the transcriptome in the testicular ECs. However, cellular senescence is thought to be a complex phenomenon involving numerous changes in gene expression and molecular signalings due to genetic or epigenetic changes. For future projects, it would be interesting to conduct several epigenetic analyses, such as ChIP-seq for histone modifications or ATAC-seq for chromatin structure. In addition, it would be required to determine in detail what types of changes occur in ECs with age and how these changes work to suppress the proliferative ability of spermatogonia by using these genetic and epigenetic analyses. In addition, we have focused only on the testicular environment in this study. Since aging is an alteration of the multisystemic environments including hormone secretions or immune systems, future studies should analyze physiological changes of the whole body, such as endocrine and/or immunology.

## STAR★Methods

### Key resources table

**Table:** 

REAGENT or RESOURCE	SOURCE	IDENTIFIER
**Antibodies**		

MVH	Abcam	Cat# ab13840; RRID: AB_443012
TRA54	BioAcademia	Cat# 73–001; RRID: AB_2455096
PLZF	Santa Cruz	Cat# sc-22839; RRID: AB_2304760
Ki67	BioLegend	Cat# 652402; RRID: AB_11203533
P21	Abcam	Cat# ab188224; RRID: AB_2734729
Histone H3	Abcam	Cat# ab1791; RRID: AB_302613
CD34	Thermo Fisher	Cat# 14-0341-82; RRID: AB_2536502
AlexaFluor488-conjugated donkey anti-rat IgG	Cell Signaling Technology	Cat# 4416; RRID: AB_10693769
AlexaFluor555-conjugated donkey anti-Rabbit IgG	Thermo Fisher	Cat# A-31572; RRID: AB_162543
HRP-conjugated anti-rabbit IgG	Cell Signaling Technology	Cat# 7074; RRID: AB_2099233
AlexaFluor555-conjugated donkey anti-Rat IgG	Abcam	Cat# ab150154; RRID: AB_2813834

**Chemicals, peptides, and recombinant proteins**		

Dasatinib	Sigma-Aldrich	SML2589
Quercetin	Wako	514–58343
Dulbecco’s Modified Eagle Medium	Nacalai	08458–45
SignalStain Antibody Diluent	Cell Signaling Technology	
ProLong Diamond Antifade Mountant	Thermo Fisher	P36965
Mitomycin C	Nacalai	20898–21
Collagenase type IV	Worthington	LS004186
Hyaluronidase	Sigma-Aldrich	H3506
DNase I	Roche	11284932001
Hanks’ balanced salt solution	Nacalai	17460–15
StemPro-34	Thermo Fisher	10639011
RIPA buffer	Nacalai	16488–34
Phenylmethylsulfonyl fluoride	Cell Signaling Technology	8553
Protease inhibitor cocktail	Nacalai	04080–24
Phosphatase inhibitor	Nacalai	07574–61

**Critical commercial assays**		

Senescence β-Galactosidase Staining Kit	Cell Signaling Technology	9860
Luminata Forte	Merck	WBLUF0100
Quant-IT RiboGreen	Invitrogen	R11490
TapeStation RNA screentape	Agilent	5067–5576
Illumina TruSeq Stranded mRNA Sample Prep Kit	Illumina	RS-122-2101
KAPA Library Quantification kits	KAPA BIOSYSTEMS	KK4854
TapeStation D1000 ScreenTape	Agilent Technologies	5067–5582
THUNDERBIRD SYBR qPCR Mix	Toyobo	QPS-201
NucleoSpin RNA Plus	Macherey-Nagel	740984.250
SuperScript VILO Master Mix	Thermo Fisher	11755050

**Deposited data**		

Endothelial cell RNA-seq data	This paper	NCBI BioProject: PRJNA990953

**Experimental models: Cell lines**		

Germline stem cells (GSC)	The current study	N/A

**Experimental models: organisms/strains**		

Mouse: C57BL/6J	Japan SLC	C57BL/6JmsSlc
Mouse: C57BL/6J	CLEA-apan	C57BL/6JJcl

**Oligonucleotides**		

Gpc3-qF: CAGCCCGGACTCAAATGGG	PrimerBank (https://pga.mgh.harvard.edu/primerbank/index.html)	31980680a1
Gpc3-qR: CAGCCGTGCTGTTAGTTGGTA	PrimerBank	31980680a1
Fn1-qF: ATGTGGACCCCTCCTGATAGT	PrimerBank	1181242a1
Fn1-qR: GCCCAGTGATTTCAGCAAAGG	PrimerBank	1181242a1
Igfbp3-qF: CCAGGAAACATCAGTGAGTCC	PrimerBank	6680385a1
Igfbp3-qR: GGATGGAACTTGGAATCGGTCA	PrimerBank	6680385a1
Myh10-qF: GGAATCCTTTGGAAATGCGAAGA	PrimerBank	33598964a1
Myh10-qR: GCCCCAACAATATAGCCAGTTAC	PrimerBank	33598964a1
Basp1-qF: GCGAGGCCAAAAAGACTGAG	PrimerBank	15076513a1
Basp1-qR: CCGCGCTGCTAGGTTTAGAG	PrimerBank	15076513a1
Cdkn2a-qF: AACTCTTTCGGTCGTACCCC	PrimerBank	1162949a1
Cdkn2a-qR: GCGTGCTTGAGCTGAAGCTA	PrimerBank	1162949a1
Gjb5_qF: TGTGGGGAGACGACCAGAA	PrimerBank	6753996a1
Gjb5_qR: CGGGATTCGGGTAAAGGTAAC	PrimerBank	6753996a1
Sorcs2_qF: CCAAGGACCTACAGATCATCAGC	PrimerBank	13562100a1
Sorcs2_qR: CAGGGAATAGCGAACCCCA	PrimerBank	13562100a1
Rarres2_qF: GCCTGGCCTGCATTAAAATGG	PrimerBank	21313658a1
Rarres2_qR: CTTGCTTCAGAATTGGGCAGT	PrimerBank	21313658a1
Fst_qF: TGCTGCTACTCTGCCAGTTC	PrimerBank	6679867a1
Fst_qR: GTGCTGCAACACTCTTCCTTG	PrimerBank	6679867a1

**Software and algorithms**		

Prism V10.0.0	GraphPad	https://www.graphpad.com/
BZ- X Viewer	Keyence	https://www.keyence.co.jp/
BZ-X Analyzer	Keyence	https://www.keyence.co.jp/
Metascape	Zhou et al.^47^	https://metascape.org/
GSEA v4.3.2		https://www.gsea-msigdb.org/gsea/index.jsp
Fiji	NIH	https://imagej.net/software/fiji/downloads

**Other**		

Bouin solution	Wako	586–70483
Lemosol A	Wako	128–04417
Hematoxylin	Muto Pure Chemical	30141
Mount-Quick	Daido Sangyo	
BZ-X700 fluorescent microscope	Keyence	https://www.keyence.co.jp/
NovaSeq	Illumina	
Luna-FL Dual Fluorescence Cell Counter	Logos biosystems	L20001
StepOne real-time PCR system	Thermo Fisher	StepOne-01

### Resource availability

#### Lead contact

Further information and requests for resources and reagents should be directed to and will be fulfilled by the lead contact, Manabu Ozawa (semi@ims.u-tokyo.ac.jp).

#### Material availability

This study did not develop any new unique reagents.

#### Data and code availability

RNA-seq data are publically available at NCBI BioProject (key resources table, NCBI BioProject: PRJNA990953). There are no restrictions on the use of these data in future research. This paper does not report original code. Any additional information required to reanalyze the data reported in this paper is available from the lead contact upon request.

### Experimental model and mouse details

#### Animals and ethics

C57BL/6J mice were purchased from Japan SLC (Shizuoka, Japan) or Clea-Japan (Tokyo, Japan). All mice were housed under pathogen-free conditions with a 12-h light/12-h dark photoperiodic cycle, and food and water were provided *ad libitum* at the experimental animal facility in the Institute of Medical Science, University of Tokyo. All mouse experiments were approved by the Institutional Animal Care and Use Committee of the University of Tokyo (approval number: A22-30) and conformed to their guidelines as well as ARRIVE guidelines (https://arriveguidelines.org).

### Method details

#### Fertility test

Male mice aged 2 months or >2 years (designated as young or aged mice, respectively) were individually housed with two 8-week-old females for 2 months. Then, the number of deliveries or litter sizes after each delivery were counted.

#### Histological analysis and sperm count

Testes were fixed in Bouin solution (Wako, Osaka, Japan) for 4 h at 4°C and then dehydrated via serial treatment with gradient ethanol solutions (starting with 70%, 80%, 90%, 95%, and 100% [v/v] ethanol, with incubation for >1 h for each step). Subsequently, these dehydrated testes were embedded in paraffin to obtain 5-μm thick sections. For histological staining, sections were deparaffinized using Lemosol A (Wako), rehydrated via serial treatment with gradient ethanol solutions (starting with 100%, 95%, 80%, and 70% [v/v] ethanol, with incubation for 5 min for each step), washed with water, and then stained with periodic acid–Schiff (PAS) stain, followed by counterstaining of the nucleus using hematoxylin (Muto Pure Chemicals Co., Tokyo, Japan). The slides were then covered using a coverslip with Mount-Quick mounting medium (Daido Sangyo, Tokyo, Japan) and observed under a microscope (BZ-X700; Keyence, Osaka, Japan). To measure the proportion of seminiferous tubules showing degeneration, >230 seminiferous tubules per individual were observed, and the degeneration ratios (one biological replicate) were calculated. We conducted this analysis with ten biological replicates in each age group. The thickness of the germ cell layer, from the outmost layer of the basal side to the innermost layer of the lumen side, was measured using Fiji (NIH). Measurements were obtained in both vertical and horizontal directions, and their average lengths represented the cell layer length of one seminiferous tubule. Moreover, we measured >50 seminiferous tubules per individual, and the resulting average value was used as one biological replicate. This analysis was performed with eight biological replicates for each age group. For sperm counting, the cauda epididymis was sliced using a pair of scissors and incubated in Dulbecco’s Modified Eagle Medium (Nacalai, Kyoto, Japan) containing 10% (v/v) fetal bovine serum (FBS) at 37°C for 30 min. After incubation, the supernatants containing sperm were diluted in the 10-fold volume of water to inhibit their mobility, after which sperm counting was performed using a hemocytometer using an inverted microscope (CKX31; Olympus, Tokyo, Japan).

#### Immunohistochemistry

For immunohistochemistry, the testes of mice of specific ages were fixed overnight at 4°C in phosphate-buffered saline containing 4% (w/v) paraformaldehyde (PFA), dehydrated, and embedded in paraffin, followed by sectioning. Subsequently, the paraffin-embedded sections were deparaffinized and rehydrated as described previously. We then boiled the rehydrated sections using sodium citrate buffer (10 mM sodium citrate and 0.05% [v/v] Tween 20 prepared in water; pH 6.0) or Tris-ethylenediaminetetraacetic acid (EDTA) buffer (10 mM Tris, 1 mM EDTA, and 0.05% [v/v] Tween 20 prepared in water; pH 9.0), via autoclaving (110°C, 20 min) to reactivate the antigens. Sections were blocked for 1 h at room temperature using Tris-buffered saline solution containing 0.1% (v/v) Tween 20, 5% (w/v) bovine serum albumin, and 10% (v/v) normal donkey serum and then exposed overnight at 4°C to primary antibodies diluted in SignalStain Antibody Diluent (Cell Signaling Technologies, Danvers, MA, USA). We used the following antibodies for immunohistochemical analysis: anti-MVH (a germ cell marker showing strong spermatocyte-specific cytoplasmid localization), TRA54 (a haploid spermatid marker), anti-PLZF (a spermatogonia marker), and Ki67 (a cell proliferation marker). Immunoreactivity was visualized using Alexa Fluor 488- or 555-conjugated host animal-specific secondary antibodies. After staining with ProLong Diamond Antifade Mountant with NucBlue Stain (Thermo Fisher Scientific), the sections were covered with a coverslip. We observed the stained sections under a microscope to detect fluorescein (Epi-fluorescent microscope, BZ-X700; Keyence, Osaka, Japan). The seminiferous tubules showing insufficient spermatocyte layers or incomplete meiosis (Figure 2A, indicated using white or yellow arrows, respectively) were counted, and their ratios with respect to the total number of seminiferous tubules were calculated.

#### Germline stem cell development and culture

We used wild-type mice (B6J background) for GSC development according to a previously reported method.^15^ Briefly, the testes of 7-day-old postpartum neonates were collected and enzymatically digested into single cells. Then, single cells were seeded into a GSC culture medium containing mitotically inactivated mouse embryonic fibroblasts (MEF; treated with 10 μg/mL mitomycin C [MMC] for 2 h before use); the medium was slightly modified.^40^ These cells were then cultured at 37°C under a humid atmosphere with 5% CO_2,_ wherein all testicular somatic cells inhibited proliferation within a short period, and stably self-renewing GSCs were developed after several passages. Subsequently, these GSCs were passaged every 7–10 days at a density of 1 × 10^5^ cells/mL on MEF.

#### Testicular EC culture and immunofluorescence

Testicular ECs were recovered from young or aged mice using previously reported methods.^18^^,^^31^ Briefly, each testis was sliced using a pair of scissors and further digested at 37°C for 30 min with agitation using a buffer solution containing collagenase type IV (1 mg/mL; Worthington, Lakewood, NJ, USA), hyaluronidase (1 mg/mL; Sigma-Aldrich), and DNase I (10 μg/mL; Roche, Tokyo, Japan). Next, the digested tissues were washed once with Hanks’ balanced salt solution. Then, the cells were seeded onto gelatin-coated dishes containing EC culture medium (50:50 mixture of alpha MEM [Nacalai, Kyoto, Japan] and StemPro-34 [Thermo Fisher Scientific]) supplemented with 20% (v/v) FBS and were cultured until specific time points for each assay. To confirm the purity of the obtained ECs, immunofluorescence was performed. In brief, cultured cells were fixed with ice-cold methanol (100%) for 5 min and then blocked with PBS containing Tween 20 (0.1%, v/v) and donkey serum (10%, v/v) for 30 min. Then, cells were exposed overnight at 4°C to an antibody against CD34 (Thermo Fisher, #14-0341-82), which is a marker of testicular ECs.^18^^,^^31^ Immunoreactivity was visualized using Alexa Fluor 555-conjugated anti-rat IgG secondary antibody. After staining with ProLong Diamond Antifade Mountant with NucBlue Stain (Thermo Fisher Scientific), the sections were covered with a coverslip. We observed the stained cells under an epi-fluorescent microscope (BZ-X700; Keyence).

#### Coculture of ECs and GSCs

Coculture of ECs and GSCs was performed as described previously.^17^^,^^18^ Briefly, ECs (1 × 10^5^ cells seeded into a gelatin-coated 35-mm dish) at 9 dph were treated with MMC (10 μg/mL) for 3 h before being used for coculture with GSCs (2 × 10^5^ GSC per dish). Coculture of ECs and GSCs was performed in GSC culture medium, and the number of GSCs at each passage was counted using the Luna-FL Dual Fluorescence Cell Counter (Logos Biosystems, Gyeonggi-do, South Korea).

#### Senescence-associated β-galactosidase staining (SA-β-gal)

Senescence β-Galactosidase Staining Kit (Cell Signaling Technology) was used for SA-β-gal staining according to the manufacturer’s instructions. For staining testis sections, testes without fixation were embedded into a 22-oxacalcitriol compound (Sakura Finetek, Tokyo, Japan), frozen in liquid nitrogen, and sliced to obtain 5-μm thick sections. The sectioned tissues were then fixed and used for SA-β-gal staining. After SA-β-gal staining, the nucleus of the section was counterstained with 4′,6-diamidino-2-phenylindole (DAPI). These stained sections were covered with a coverslip and observed under a microscope (BZ-X700, Keyence). For *in vitro* EC staining, ECs were seeded into a gelatin-coated culture dish containing EC medium until 9 dph, which were then fixed and used for SA-β-gal staining. Next, we stained the nucleus with DAPI and observed the ECs under a microscope (BZ-X700, Keyence). The number of SA-β-gal–positive cells was counted manually using Fiji cell counter plug-in (ImageJ, NIH). The number of DAPI-positive cells in the same field was counted using BZ-X Analyzer (Keyence).

#### Western blot analysis

We collected ECs that were cultured for 9 dph. Then, we extracted total protein via sonication using RIPA buffer (Nacalai, Kyoto, Japan) containing phenylmethylsulfonyl fluoride (Cell Signaling Technology, Danvers, MA, USA), protease inhibitor cocktail (Nacalai), and phosphatase inhibitor (Nacalai). The extracted protein lysate was analyzed via the standard western blotting protocol. An animal-specific secondary antibody conjugated to horseradish peroxidase was used to visualize each protein via Luminata Forte (Merck Millipore). The protein signal was detected using the ChemiDoc Imaging System (Bio-Rad, Hercules, CA, USA). Figure S6 presents all raw membrane images.

#### RNA-seq

We collected ECs that were cultured for 9 dph via trypsinization and used them for RNA-seq analysis. Total RNA concentration was calculated using Quant-IT RiboGreen (Invitrogen, #R11490). To assess the integrity of total RNA, we ran the samples on the TapeStation RNA screentape (Agilent, #5067–5576). Only high-quality RNA samples with an integrity number of >7.0 were used for RNA library construction. A library was independently constructed using 1 μg of total RNA for each sample via Illumina TruSeq Stranded mRNA Sample Prep Kit (Illumina, Inc., San Diego, CA, USA, #RS-122-2101). We first purified poly-A–containing mRNA molecules using poly-T–attached magnetic beads, fragmented the mRNA samples into small pieces using divalent cations at elevated temperatures, copied the cleaved RNA fragments into first-strand cDNA using SuperScript II reverse transcriptase (Invitrogen, #18064014) and random primers, and then synthesized second-strand cDNA using DNA Polymerase I, RNase H, and dUTP. Subsequently, these cDNA fragments underwent an end-repair process, wherein a single “A” base was added and the adapters were ligated. The products were then purified and subjected to PCR to construct the final cDNA library. The libraries were quantified using KAPA Library Quantification kits for Illumina Sequencing platforms according to the qPCR Quantification Protocol Guide (KAPA BIOSYSTEMS, #KK4854) and qualified using the TapeStation D1000 ScreenTape (Agilent Technologies, # 5067–5582). Next, the indexed libraries were submitted to Illumina NovaSeq (Illumina, Inc., San Diego, CA, USA), followed by paired-end (2 × 100 bp) sequencing by Macrogen Inc.

#### Quantification of mRNA expression using real-time PCR

Total RNA was extracted from cultured GSCs using NucleoSpin RNA Plus (Macherey-Nagel, Düren, Germany) following the manufacturer’s instructions, which included DNase treatment as part of the RNA extraction procedure. The total RNA was used to synthesize cDNA in a reaction volume of 10 μL via SuperScript VILO (Thermo Fisher) according to the manufacturer’s instructions. The synthesized cDNA was used for quantitative real-time PCR analysis (StepOne, Thermo Fisher) in a 10-μL reaction mixture of containing 1× THUNDERBIRD SYBER qPCR Mix (Toyobo, Osaka, Japan) and 0.3 μM each of forward and reverse primers. The fold difference was calculated using the ΔΔCt method, with GAPDH as the reference gene.

### Volcano plot and enrichment analysis

The volcano plot was constructed based on log2 fold change and −log10 values (p-value). Enrichment analysis of GO terms and KEGG pathways in DEGs between young and aged ECs was conducted using Metascape.^47^ For GO and KEGG enrichment analyses, we considered significant DEGs with a log2 fold change of ≥0.5 or ≤ −0.5, and p-values of <0.05. Additionally, GSEA was performed using the hallmark gene set collection from MSigDB via GSEA v4.3.2 (https://www.gsea-msigdb.org/gsea/index.jsp), as previously described.^48^

#### Heatmap

A heatmap was constructed using Z-score–normalized fragments per kilobase per million values that were clustered hierarchically using the Ward’s method.

#### Senolytic treatments

ECs were treated with dasatinib (50 nM; Sigma-Aldrich, St. Louis, MO, USA) and quercetin (25 μM, Wako) (D + Q) for 5 days from 4 to 9 dph. Then, the treated cells were subjected to SA-β-gal staining or GSC coculture.

### Quantification and statistical analysis

#### Statistical analysis

All numerical data are expressed as the mean ± SEM of three or more independent replications (the exact value of n in each experiment is presented in Figure Legends). Differences between ages or treatments were analyzed using the two-tailed Student’s *t* test, except for the number of fertile males of different ages in which χ^2^ analysis was used for comparison. p-values of <0.05 were considered to indicate statistical significance. Statistical analyses were performed using Prism V10.0.0 (GraphPad).

### Additional resources

This study did not contain any additional resources.

## References

[1] May-Panloup P., Boucret L., Chao de la Barca J.M., Desquiret-Dumas V., Ferré-L'Hotellier V., Morinière C., Descamps P., Procaccio V., Reynier P. (2016). Ovarian ageing: the role of mitochondria in oocytes and follicles. Hum. Reprod. Update.

[2] Lee Y., Bohlin J., Page C.M., Nustad H.E., Harris J.R., Magnus P., Jugessur A., Magnus M.C., Håberg S.E., Hanevik H.I. (2022). Associations between epigenetic age acceleration and infertility. Hum. Reprod..

[3] Greaney J., Wei Z., Homer H. (2018). Regulation of chromosome segregation in oocytes and the cellular basis for female meiotic errors. Hum. Reprod. Update.

[4] Sher G., Keskintepe L., Keskintepe M., Ginsburg M., Maassarani G., Yakut T., Baltaci V., Kotze D., Unsal E. (2007). Oocyte karyotyping by comparative genomic hybridization [correction of hybrydization] provides a highly reliable method for selecting "competent" embryos, markedly improving in vitro fertilization outcome: a multiphase study. Fertil. Steril..

[5] Di Emidio G., Falone S., Vitti M., D'Alessandro A.M., Vento M., Di Pietro C., Amicarelli F., Tatone C. (2014). SIRT1 signalling protects mouse oocytes against oxidative stress and is deregulated during aging. Hum. Reprod..

[6] Kidd S.A., Eskenazi B., Wyrobek A.J. (2001). Effects of male age on semen quality and fertility: a review of the literature. Fertil. Steril..

[7] Johnson S.L., Dunleavy J., Gemmell N.J., Nakagawa S. (2015). Consistent age-dependent declines in human semen quality: a systematic review and meta-analysis. Ageing Res. Rev..

[8] Toriello H.V., Meck J.M., Professional Practice and Guidelines Committee (2008). Professional Practice and Guidelines Committee. Statement on guidance for genetic counseling in advanced paternal age. Genet. Med..

[9] Mazur D.J., Lipshultz L.I. (2018). Infertility in the Aging Male. Curr. Urol. Rep..

[10] Ryu B.Y., Orwig K.E., Oatley J.M., Avarbock M.R., Brinster R.L. (2006). Effects of aging and niche microenvironment on spermatogonial stem cell self-renewal. Stem Cell..

[11] Zhang X., Ebata K.T., Robaire B., Nagano M.C. (2006). Aging of male germ line stem cells in mice. Biol. Reprod..

[12] Hasegawa K., Saga Y. (2012). Retinoic acid signaling in Sertoli cells regulates organization of the blood-testis barrier through cyclical changes in gene expression. Development.

[13] Hasegawa K., Namekawa S.H., Saga Y. (2013). MEK/ERK signaling directly and indirectly contributes to the cyclical self-renewal of spermatogonial stem cells. Stem Cell..

[14] Kanatsu-Shinohara M., Yamamoto T., Toh H., Kazuki Y., Kazuki K., Imoto J., Ikeo K., Oshima M., Shirahige K., Iwama A. (2019). Aging of spermatogonial stem cells by Jnk-mediated glycolysis activation. Proc. Natl. Acad. Sci. USA.

[15] Kanatsu-Shinohara M., Ogonuki N., Inoue K., Miki H., Ogura A., Toyokuni S., Shinohara T. (2003). Long-term proliferation in culture and germline transmission of mouse male germline stem cells. Biol. Reprod..

[16] Kanatsu-Shinohara M., Shinohara T. (2013). Spermatogonial stem cell self-renewal and development. Annu. Rev. Cell Dev. Biol..

[17] Bhang D.H., Kim B.J., Kim B.G., Schadler K., Baek K.H., Kim Y.H., Hsiao W., Ding B.S., Rafii S., Weiss M.J. (2018). Testicular endothelial cells are a critical population in the germline stem cell niche. Nat. Commun..

[18] Kitadate Y., Jörg D.J., Tokue M., Maruyama A., Ichikawa R., Tsuchiya S., Segi-Nishida E., Nakagawa T., Uchida A., Kimura-Yoshida C. (2019). Competition for Mitogens Regulates Spermatogenic Stem Cell Homeostasis in an Open Niche. Cell Stem Cell.

[19] Gow A., Southwood C.M., Li J.S., Pariali M., Riordan G.P., Brodie S.E., Danias J., Bron- stein J.M., Kachar B., Lazzarini R.A. (1999). CNS myelin and sertoli cell tight junction strands are absent in Osp/claudin-11 null mice. Cell.

[20] Wu X., Peppi M., Vengalil M.J., Maheras K.J., Southwood C.M., Bradley M., Gow A. (2012). Transgene-mediated rescue of spermatogenesis in Cldn11-null mice. Biol. Reprod..

[21] Dong Y., Zhang L., Bai Y., Zhou H.M., Campbell A.M., Chen H., Yong W., Zhang W., Zeng Q., Shou W., Zhang Z.Y. (2014). Phosphatase of regenerating liver 2 (PRL2) deficiency impairs Kit signaling and spermatogenesis. J. Biol. Chem..

[22] Minamino T., Orimo M., Shimizu I., Kunieda T., Yokoyama M., Ito T., Nojima A., Nabetani A., Oike Y., Matsubara H. (2009). A crucial role for adipose tissue p53 in the regulation of insulin resistance. Nat. Med..

[23] Omori S., Wang T.W., Johmura Y., Kanai T., Nakano Y., Kido T., Susaki E.A., Nakajima T., Shichino S., Ueha S. (2020). Generation of a p16 Reporter Mouse and Its Use to Characterize and Target p16high Cells In Vivo. Cell Metabol..

[24] Johmura Y., Yamanaka T., Omori S., Wang T.W., Sugiura Y., Matsumoto M., Suzuki N., Kumamoto S., Yamaguchi K., Hatakeyama S. (2021). Senolysis by glutaminolysis inhibition ameliorates various age-associated disorders. Science.

[25] van der Loo B., Fenton M.J., Erusalimsky J.D. (1998). Cytochemical detection of a senescence-associated beta-galactosidase in endothelial and smooth muscle cells from human and rabbit blood vessels. Exp. Cell Res..

[26] Price J.S., Waters J.G., Darrah C., Pennington C., Edwards D.R., Donell S.T., Clark I.M. (2002). The role of chondrocyte senescence in osteoarthritis. Aging Cell.

[27] Debacq-Chainiaux F., Erusalimsky J.D., Campisi J., Toussaint O. (2009). Protocols to detect senescence-associated beta-galactosidase (SA-betagal) activity, a biomarker of senescent cells in culture and in vivo. Nat. Protoc..

[28] Cohen C., Le Goff O., Soysouvanh F., Vasseur F., Tanou M., Nguyen C., Amrouche L., Le Guen J., Saltel-Fulero O., Meunier T. (2021). Glomerular endothelial cell senescence drives age-related kidney disease through PAI-1. EMBO Mol. Med..

[29] Sampaio Gonçalves D., Keyes W.M. (2022). Endothelial cells give a boost to senescence surveillance. Genes Dev..

[30] Bloom S.I., Islam M.T., Lesniewski L.A., Donato A.J. (2023). Mechanisms and consequences of endothelial cell senescence. Nat. Rev. Cardiol..

[31] Seandel M., James D., Shmelkov S.V., Falciatori I., Kim J., Chavala S., Scherr D.S., Zhang F., Torres R., Gale N.W. (2007). Generation of functional multipotent adult stem cells from GPR125+ germline progenitors. Nature.

[32] He S., Sharpless N.E. (2017). Senescence in Health and Disease. Cell.

[33] Coppé J.P., Desprez P.Y., Krtolica A., Campisi J. (2010). The senescence-associated secretory phenotype: the dark side of tumor suppression. Annu. Rev. Pathol..

[34] Palmer A.K., Xu M., Zhu Y., Pirtskhalava T., Weivoda M.M., Hachfeld C.M., Prata L.G., van Dijk T.H., Verkade E., Casaclang-Verzosa G. (2019). Targeting senescent cells alleviates obesity-induced metabolic dysfunction. Aging Cell.

[35] Zhu Y., Tchkonia T., Pirtskhalava T., Gower A.C., Ding H., Giorgadze N., Palmer A.K., Ikeno Y., Hubbard G.B., Lenburg M. (2015). The Achilles' heel of senescent cells: from transcriptome to senolytic drugs. Aging Cell.

[36] Lewis-McDougall F.C., Ruchaya P.J., Domenjo-Vila E., Shin Teoh T., Prata L., Cottle B.J., Clark J.E., Punjabi P.P., Awad W., Torella D. (2019). Aged-senescent cells contribute to impaired heart regeneration. Aging Cell.

[37] Roos C.M., Zhang B., Palmer A.K., Ogrodnik M.B., Pirtskhalava T., Thalji N.M., Hagler M., Jurk D., Smith L.A., Casaclang-Verzosa G. (2016). Chronic senolytic treatment alleviates established vasomotor dysfunction in aged or atherosclerotic mice. Aging Cell.

[38] Kim S.R., Puranik A.S., Jiang K., Chen X., Zhu X.Y., Taylor I., Khodadadi-Jamayran A., Lerman A., Hickson L.J., Childs B.G. (2021). Progressive Cellular Senescence Mediates Renal Dysfunction in Ischemic Nephropathy. J. Am. Soc. Nephrol..

[39] Lucio R.A., Tlachi-López J.L., Eguibar J.R., Ågmo A. (2013). Sperm count and sperm motility decrease in old rats. Physiol. Behav..

[40] Ozawa M., Fukuda T., Sakamoto R., Honda H., Yoshida N. (2016). The histone demethylase FBXL10 regulates the proliferation of spermatogonia and ensures long-term sustainable spermatogenesis in mice. Biol. Reprod..

[41] Senoo M., Takijiri T., Yoshida N., Ozawa M., Ikawa M. (2019). PTBP1 contributes to spermatogenesis through regulation of proliferation in spermatogonia. J. Reprod. Dev..

[42] Nie X., Munyoki S.K., Sukhwani M., Schmid N., Missel A., Emery B.R., Donor C., Stukenborg J.B., Mayerhofer A., Orwig K.E. (2022). Single-cell analysis of human testis aging and correlation with elevated body mass index. Dev. Cell.

[43] Sperka T., Wang J., Rudolph K.L. (2012). DNA damage checkpoints in stem cells, ageing and cancer. Nat. Rev. Mol. Cell Biol..

[44] López-Otín C., Blasco M.A., Partridge L., Serrano M., Kroemer G. (2023). Hallmarks of aging: An expanding universe. Cell.

[45] Ferreiro M.E., Méndez C.S., Glienke L., Sobarzo C.M., Ferraris M.J., Pisera D.A., Lustig L., Jacobo P.V., Theas M.S. (2023). Unraveling the effect of the inflammatory microenvironment in spermatogenesis progression. Cell Tissue Res..

[46] Ferreiro M.E., Amarilla M.S., Glienke L., Méndez C.S., González C., Jacobo P.V., Sobarzo C.M., De Laurentiis A., Ferraris M.J., Theas M.S. (2019). The inflammatory mediators TNFα and nitric oxide arrest spermatogonia GC-1 cell cycle. Reprod. Biol..

[47] Zhou Y., Zhou B., Pache L., Chang M., Khodabakhshi A.H., Tanaseichuk O., Benner C., Chanda S.K. (2019). Metascape provides a biologist-oriented resource for the analysis of systems-level datasets. Nat. Commun..

[48] Subramanian A., Tamayo P., Mootha V.K., Mukherjee S., Ebert B.L., Gillette M.A., Paulovich A., Pomeroy S.L., Golub T.R., Lander E.S., Mesirov J.P. (2005). Gene set enrichment analysis: a knowledge-based approach for interpreting genome-wide expression profiles. Proc. Natl. Acad. Sci. USA.

