# Supplementary material for: Age-related decline in spermatogenic activity accompanied with endothelial cell senescence in male mice
Source: iScience. 2023 Nov 13;26(12):108456. doi: 10.1016/j.isci.2023.108456 (PMC10700819; doi:10.1016/j.isci.2023.108456)
Supplement: Document S1. Figures S1–S6 [file mmc1.pdf]

iScience, Volume *26*

Supplemental information

Age-related decline in spermatogenic activity accompanied with endothelial cell senescence in male mice

Manabu Ozawa, Hideto Mori, Tsutomu Endo, Yu Ishikawa-Yamauchi, Daisuke Motooka, Chihiro Emori, and Masahiro Ikawa

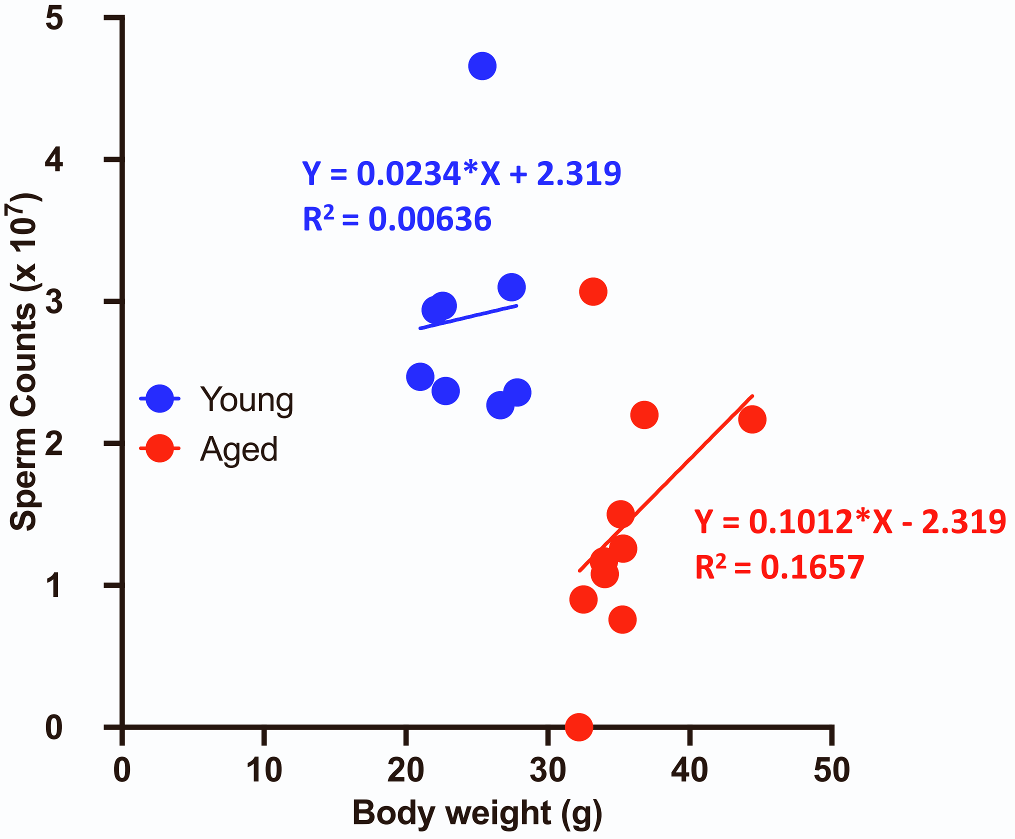

Supplemental Figure S1. ***Dot plot showing body weight and sperm count in individual males. Related to Figure 1.***

The x-axis represents body weight (g) and the y-axis represents sperm count (x 10^7). The dots represent biological replicates of individual males (n = 8, young group; n = 10, aged group).

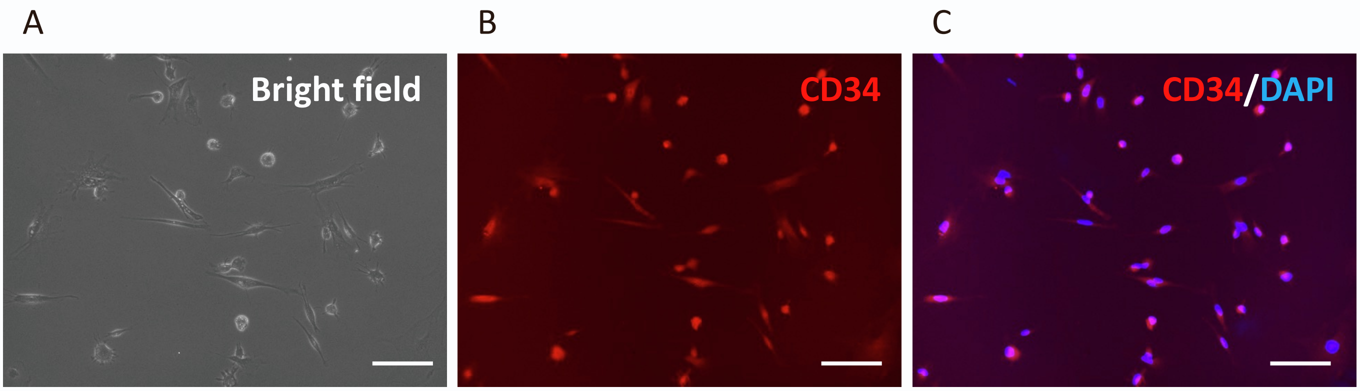

Supplemental Figure S2. ***Immunofluorescence evaluation of isolated endothelial cells. Related to Figure 3.***

Isolated and cultivated testicular cells of the same field of (A) blight field, (B) stained with anti-CD34 antibody, or (C) merged image of CD34 and DAPI. The scale bar denotes 100 μm.

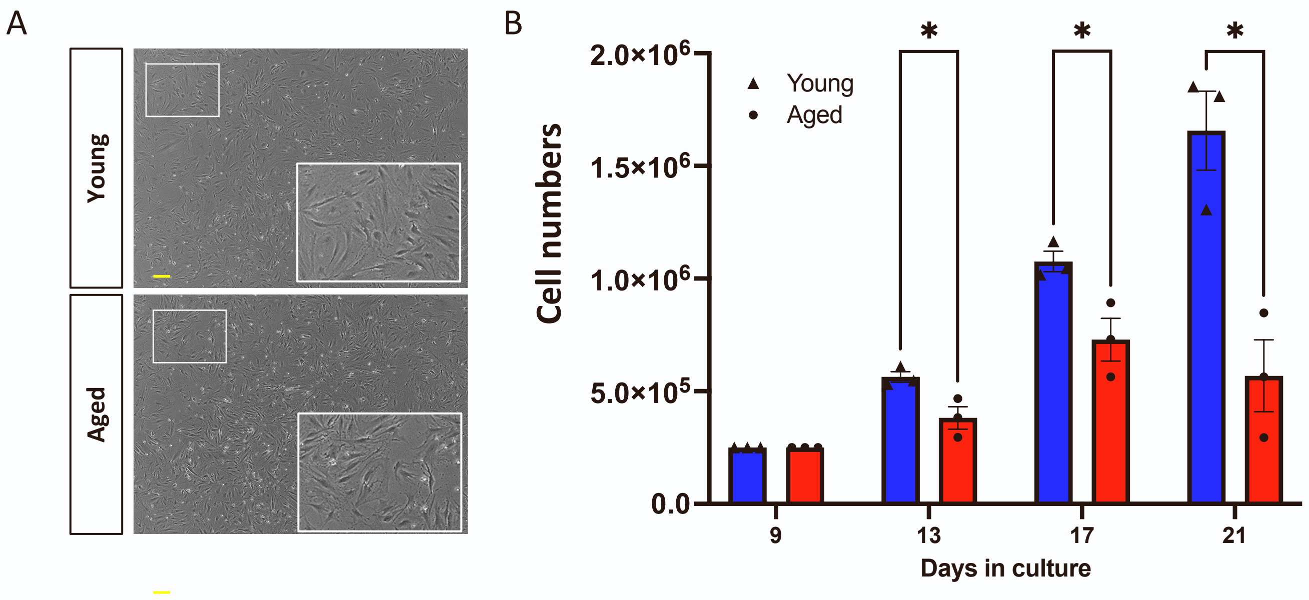

Supplemental Figure S3. ***In vitro culture of testicular endothelial cells. Related to Figure 3.***

(A) Representative morphologies of testicular EC in vitro. The scale bar denotes 200 μm.

(B) In vitro growing speed of ECs from the young and aged mice. The dots represent biological replicates of individual males (n = 3, young group; n = 3, aged group). The asterisk depicts a significant difference ($P < 0.05$).

Supplemental Fig. S4

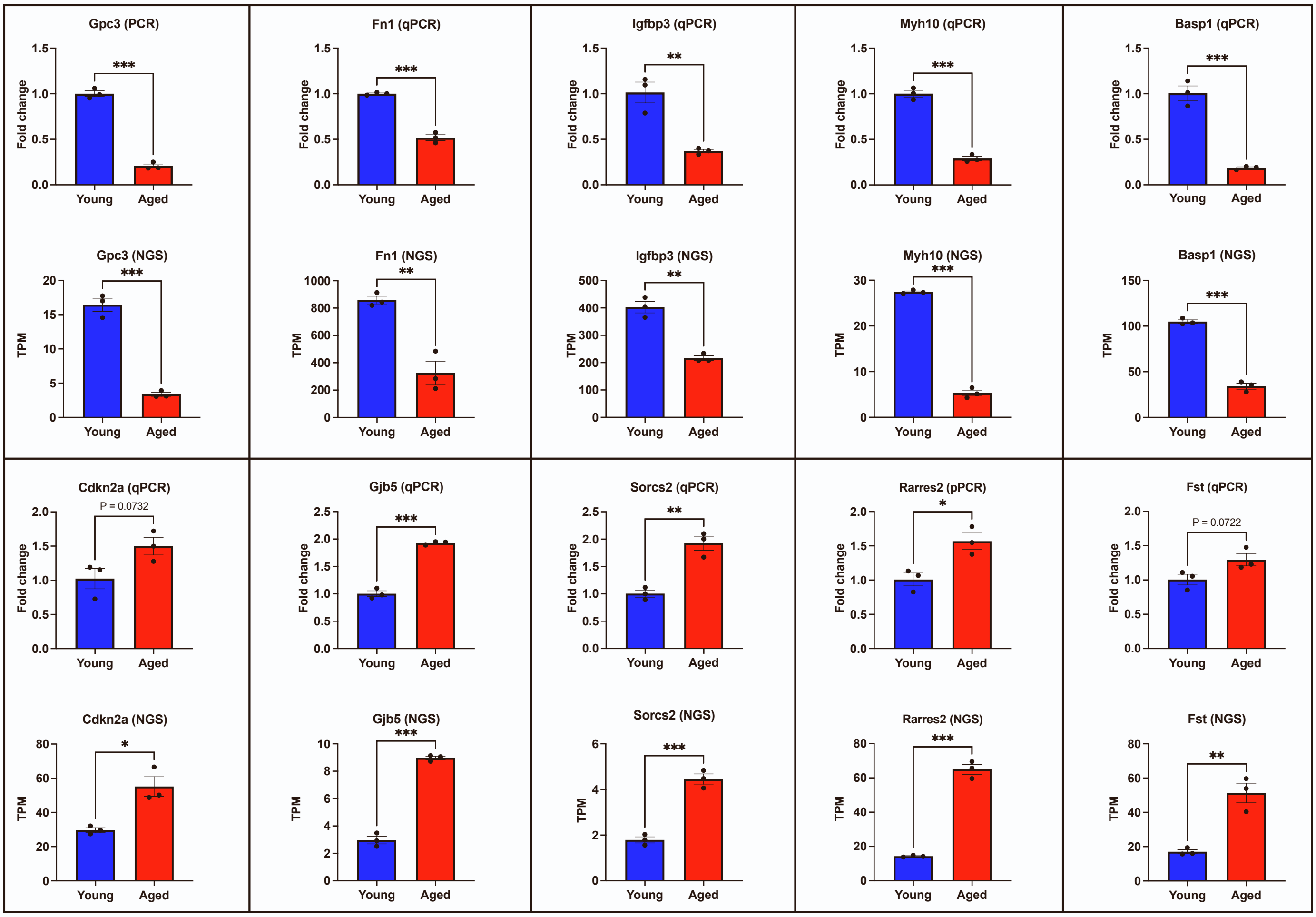

Supplemental Figure S4. ***Validation of RNA-seq data in the ECs by quantitative RT-PCR. Related to Figure 4.***

The upper row in the square shows the qPCR results, and the lower row shows the RNA-seq results. The dots represent biological replicates of individual males (n = 3, young group; n = 3, aged group). *P < 0.05, **P < 0.01, or ***P < 0.001, indicating a significant difference.

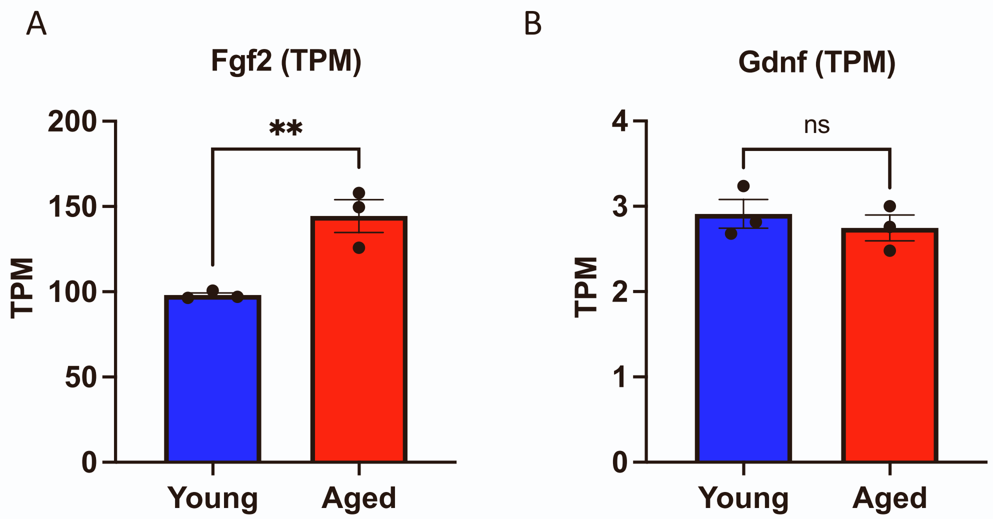

Supplemental Figure S5. ***Expression levels of Fgf2 and Gdnf in EC of young or aged mice. Related to Figure 4.***

(A) TPM of Fgf2 in testicular ECs from each age. The dots represent biological replicates of individual males (n = 3, young group; n = 3, aged group). The asterisk depicts a significant difference (P < 0.01).

(B) TPM of Gdnf in testicular ECs from each age. The dots represent biological replicates of individual males (n = 3, young group; n = 3, aged group)..

A

P21

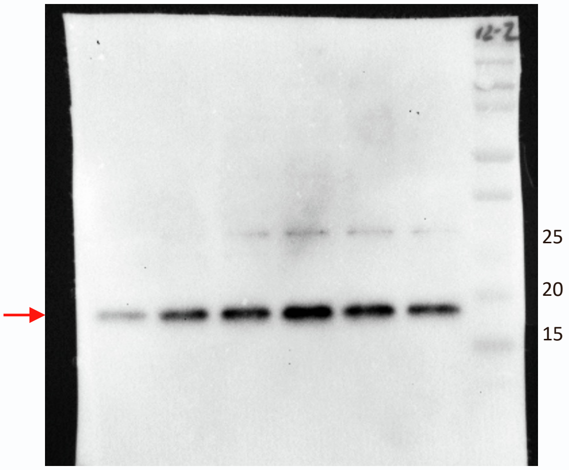

B

Histone H3

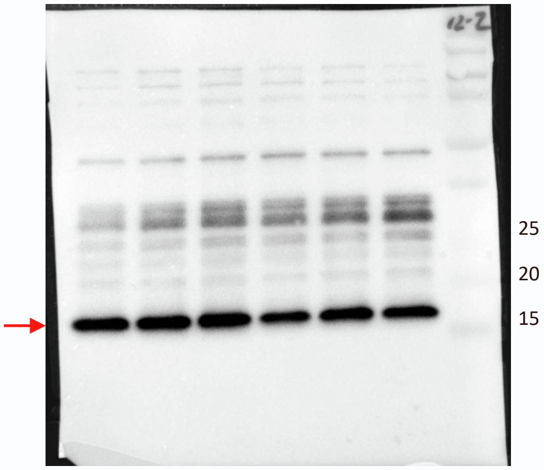

Supplemental Figure S6. ***Original membrane images of Western Blotting. Related to Figure 3.***
(A and B) Original membrane images presented in Figure 1D.
